# Enhanced Deep Learning Architectures for Face Liveness Detection for Static and Video Sequences

**DOI:** 10.3390/e22101186

**Published:** 2020-10-21

**Authors:** Ranjana Koshy, Ausif Mahmood

**Affiliations:** Computer Science and Engineering Department, University of Bridgeport, Bridgeport, CT 06604, USA; mahmood@bridgeport.edu

**Keywords:** face liveness detection, diffusion, SCNN, Inception v4, CNN-LSTM, Replay-Attack dataset, Replay-Mobile dataset

## Abstract

Face liveness detection is a critical preprocessing step in face recognition for avoiding face spoofing attacks, where an impostor can impersonate a valid user for authentication. While considerable research has been recently done in improving the accuracy of face liveness detection, the best current approaches use a two-step process of first applying non-linear anisotropic diffusion to the incoming image and then using a deep network for final liveness decision. Such an approach is not viable for real-time face liveness detection. We develop two end-to-end real-time solutions where nonlinear anisotropic diffusion based on an additive operator splitting scheme is first applied to an incoming static image, which enhances the edges and surface texture, and preserves the boundary locations in the real image. The diffused image is then forwarded to a pre-trained Specialized Convolutional Neural Network (SCNN) and the Inception network version 4, which identify the complex and deep features for face liveness classification. We evaluate the performance of our integrated approach using the SCNN and Inception v4 on the Replay-Attack dataset and Replay-Mobile dataset. The entire architecture is created in such a manner that, once trained, the face liveness detection can be accomplished in real-time. We achieve promising results of 96.03% and 96.21% face liveness detection accuracy with the SCNN, and 94.77% and 95.53% accuracy with the Inception v4, on the Replay-Attack, and Replay-Mobile datasets, respectively. We also develop a novel deep architecture for face liveness detection on video frames that uses the diffusion of images followed by a deep Convolutional Neural Network (CNN) and a Long Short-Term Memory (LSTM) to classify the video sequence as real or fake. Even though the use of CNN followed by LSTM is not new, combining it with diffusion (that has proven to be the best approach for single image liveness detection) is novel. Performance evaluation of our architecture on the REPLAY-ATTACK dataset gave 98.71% test accuracy and 2.77% Half Total Error Rate (HTER), and on the REPLAY-MOBILE dataset gave 95.41% accuracy and 5.28% HTER.

## 1. Introduction

Face recognition is a popular biometric authentication technique that is extensively used in many security and online systems. It has the advantages of easy deployment and being non-intrusive when compared to other biometric authentication schemes. Current approaches to face recognition have the key disadvantage of being easily spoofed where an impostor can present a photograph or recorded video of another person to the camera. Hence, face liveness detection is a crucial preprocessing step before performing the face authentication via face recognition. Various approaches have been proposed for face liveness detection on static images, such as analysis of texture differences between live and fake faces as in Reference [1], motion analysis, and deep Convolutional Neural Network (CNN) architectures, etc. Most of the recent research resulting in high accuracy face liveness detection and has focused on a two-step process of performing a speed diffusion, which is followed either by a Support Vector Machine (SVM) as a classifier [2], or a deep CNN architecture [3,4]. Existing approaches that have been proposed to address dynamic face spoofing attacks on recorded videos are based on methods such as texture analysis, motion analysis, image quality, and 3D structure information. Recent research has focused on using deep CNN architectures and recurrent neural networks (RNN) for face liveness detection on video frames.

We present real-time solutions for face liveness detection on static images by integrating anisotropic diffusion for converting the captured image to the diffused form, and the deep CNN into a single framework. The anisotropic diffusion allows the illumination energies to diffuse slowly on a uniform 2D surface, while moving faster on a 3D live face because of its non-uniformity [2]. The diffused image is then fed to the deep CNN architectures, i.e., Specialized Convolutional Neural Network (SCNN) and Inception v4. In the real-time solution using the SCNN, we compute the smoothness of diffusion parameter alpha, whereas, for the Inception v4, we fix a value for the alpha. We evaluate the performance of our proposed methods on the Replay-Attack dataset and the Replay-Mobile dataset, respectively. For the Inception v4, we experiment with various values of the parameter alpha that defines the smoothness of diffusion. On average, an alpha value of up to 75 gave better classification results in the integrated environment, since higher values blur out important information from the image. We further perform a comparison of our proposed approaches with previous state-of-the-art approaches as well as other deep architectures for face liveness detection on static images, and determine that our end-to-end approaches produce competitive accuracy with the advantage of being “real-time” on a standard medium power computer in the detection of liveness of images to counteract face spoofing.

For face liveness detection on video sequences, we develop a CNN-LSTM architecture where nonlinear diffusion is first applied to the individual frames in the sequence, and then the deep CNN and LSTM capture the deep spatial and temporal features in the sequence. Even though use of CNN followed by LSTM has been reported in the literature, our contribution is to further add anisotropic diffusion in the beginning. We evaluate the performance of the proposed video frame based face liveness framework on the Replay-Attack dataset and the Replay-Mobile dataset. We also perform a comparison of our proposed approach with previous state-of-the-art approaches for face liveness detection on video frames, and demonstrate that our architecture is very competitive in liveness detection of video sequences to counteract face spoofing. The performance of the proposed framework yields competitive results. We obtained better results compared to the reported results in the literature with the Replay-Mobile dataset, and second best results with the Replay-Attack dataset.

The rest of the paper is organized as follows. Section 2 discusses previous work related to face liveness detection on static images and video sequences. Our proposed real-time methods of integrating anisotropic diffusion and CNN architectures for liveness detection on static images, and the method of applying diffusion followed by a CNN-LSTM for liveness detection on video sequences, are discussed in Section 3. Section 4 presents a performance evaluation of our architectures on the Replay-Attack dataset and Replay-Mobile dataset, respectively, for face anti-spoofing. The concluding remarks are mentioned in Section 5.

## 2. Related Work

Many methods have been proposed by researchers for determining the liveness of a captured static image by extracting the features from a 2D image, and then feeding these to a classifier. Some of these include extraction of variations of Local Binary Patterns, and, using a Support Vector Machine (SVM) classifier to identify whether the face is real or fake. Parveen et al. [1] introduced a texture descriptor known as Dynamic Local Ternary Pattern (DLTP), where the textural properties of facial skin were explored using dynamic threshold setting, and the SVM with linear kernel was used for classification. The method proposed by Kim et al. [2] is based on the idea of differencing in surface properties between live and fake faces by using diffusion speed. They computed the diffusion speed by utilizing the total variation flow and extracted anti-spoofing features based on the local patterns of diffusion speeds, which were then fed to a linear SVM classifier for determining the liveness of the facial image. Gragnaniello et al. [5] proposed a domain-aware CNN architecture by adding appropriate regularization terms to the loss function. In the work proposed by Das et al. [6], hand-crafted features and deep features were extracted by using a combination of a Local Binary Pattern (LBP) and a pre-trained CNN model based on the VGG-16 network architecture for the liveness detection.

Some of the recent work in face liveness detection has focused on the use of deep CNN architectures [3,4,7,8], since these provide better liveness detection accuracy than the above-mentioned approaches. Rehman et al. [7] employed data randomization on small mini batches for the training of deep CNNs for liveness detection. In the proposed work by Alotaibi et al. [3], a combination of diffusion of the captured image followed by only a simple three-layer CNN architecture was utilized. The research proposed by Koshy et al. [4] used a combination of nonlinear diffusion and explored three architectures such as CNN-5, ResNet50, and Inception v4, and the best architecture was determined to be the Inception v4. The main drawback of the approaches in References [3,4] has been a requirement of a preprocessing step to obtain the diffused image before feeding it to a deep CNN for classification, making it unsuitable for real-time deployment. A part of our work in this paper enhances the ideas of References [3,4] in a better integrated approach.

Various approaches have been proposed to address dynamic face spoofing attacks to determine the liveness of a video sequence. Wang et al. [9] proposed a detection approach where the sparse structure information in 3D space was analyzed. Facial landmarks were detected from the given face video, and key frames were selected from which the sparse 3D facial structure was recovered. The structures were then aligned and the structure features were extracted for classification using an SVM classifier. Another technique reported by Anjos et al. [10] is based on foreground and background motion correlation using optical flow, where they detected motion correlations between the head of the user and the background, and then fed extracted features to a binary classifier to classify the sequence as real or fake. Wen et al. [11] proposed a detection algorithm based on Image Distortion Analysis (IDA). They extracted four different features, namely specular reflection, chromatic moment, blurriness, and color diversity, to form the feature vector, which was then fed to an SVM classifier.

A method based on the Local Binary Patterns from Three Orthogonal Planes (LBP-TOP) operator combining both space and time information into a single multi-resolution texture descriptor was presented by de Freitas Pereira et al. [12]. The histograms were computed from the local binary patterns and concatenated for classification using Linear Discriminant Analysis (LDA) and SVM. Bharadwaj et al. [13] used motion magnification followed by two approaches, where one involves texture analysis using LBP and an SVM classifier, and the other involves a motion estimation approach using a Histogram of Oriented Optical Flow (HOOF) descriptor with LDA for classification. Tang et al. [14] proposed a challenge-response liveness detection protocol called face flashing that flashes randomly generated colors and verifies the reflected light. This was repeated many times so that enough responses could be collected to ensure security. Yeh at al. [15] proposed an approach against face spoofing attacks based on perpetual image quality assessment with multi-scale analysis. They used a combination of an image quality evaluator and a quality assessment model for selecting effective pixels to create the image quality features for liveness detection. Pan et al. [16] presented a time-based presentation attack detection algorithm for capturing the texture changes in a frame sequence. They used a Motion History Image (MHI) descriptor to get the primary features, and used LBP and a pre-trained CNN to get the secondary feature vectors, which were then fed to a classifier network. In the work proposed by Asim et al. [17], LBP-TOP is cascaded with a CNN to extract spatio-temporal features from video sequences, which is followed by SVM with Radial Basis Function (RBF) kernel for classification.

Xu et al. [18] proved that a deep architecture combining LSTM with CNN can be used for face anti-spoofing in videos. Local and dense features were extracted by the CNN, while the LSTM captured the temporal relationships in the input sequences. Tu et al. [19] also proposed a joint CNN-LSTM network for face anti-spoofing in video sequences by focusing on the motion cues across video frames. They used the Eulerian motion magnification as preprocessing to enhance the facial expressions of individuals. Then the CNN was used for extracting the highly discriminative features of video frames, and the LSTM was used to capture the temporal dynamics in the videos. Costa-Pazo et al. [20] introduced the Replay-Mobile database, and they describe two face presentation attack detection methods that were applied to the database. In one method, Image Quality Measures (IQM) were used as features, and, in the other, a texture-based approach using Gabor-jets were used. Classification was done using the support vector machine with a radial basis function kernel.

Nikisins et al. [21] proposed an anomaly detection, or a one-class classifier-based face presentation attack detection system having better generalization properties against unseen types of attacks. They used an aggregated database consisting of three publicly available datasets, which includes the Replay-Attack dataset and Replay-Mobile dataset, for their experiments. Their system consisted of a preprocessor, feature extractor, and one-class classifier. They also evaluated and reported the results of some of the successful, previously published face liveness detection systems on the Replay-Attack and Replay-Mobile datasets, such as the LBP-based system in Reference [22]. The IQM-based system where feature vector of a frame is a concatenation of quality measures introduced in References [11,23], and the motion-based approach in Reference [24]. Evaluation using the LBP-based system and IQM-based system were done on the frame-level, whereas the motion-based approach was done on video sequences. Fatemifar et al. [25] adopted the anomaly detection approach where the detector is trained on genuine accesses only, using one-class classifiers built using representations obtained from deep pre-trained CNN models. Each frame in a video clip was photometrically normalized based on the retina method for reducing the impact of various lighting conditions before they were fed to pre-trained networks. They used different CNN architectures and anomaly detectors of which the class-specific Mahalanobis distance with GoogleNet features achieved better performance on the Replay-Mobile dataset. Arashloo [26] presented a one-class novelty detection approach based on kernel regression using the Replay-Mobile dataset in which only bona fide samples were used in the training process, as in Reference [25]. A projection function defined in terms of kernel regression maps bona fide samples onto a compact cluster of target samples, and provides the best separability of normal samples from outliers with classification based on the Fisher criterion. Other mechanisms, which include a multiple kernel fusion approach, sparse regularization, and client-specific and probabilistic modeling were also incorporated to improve performance.

The methods proposed by de Freitas Pereira et al. [12] and Bharadwaj et al. [13] made use of hand-crafted feature extraction, while we are using a CNN that does the feature extraction by itself, which eliminates hand-engineered feature extraction. Though the method proposed by Tu et al. [19] gave state-of-the-art performance, compared to the 13 convolution layers of the VGG-16 they used, we are using only a 4-layer CNN as the front-end of our architecture with which we get very competitive results. The work presented by Fatemifar et al. [25] and Arashloo [26] used pre-trained models such as GoogleNet, ResNet50, etc., while we designed a CNN-LSTM architecture with which we achieved better results on the Replay-Mobile dataset. The enhancement in our work is to apply nonlinear diffusion to the frames in the sequence to obtain the sharp edges and preserve the boundary locations, and then feed the diffused frames to the CNN-LSTM.

## 3. Proposed Method

For the end-to-end real-time solution for liveness detection on static images, we propose a solution similar to what was done in References [3,4]. However, instead of using a preprocessing step (via Matlab code) for diffusing the images and feeding them to the deep CNN network, we provide an end-to-end solution with diffusion as well as more advanced deep CNNs. In our framework, we use a combined architecture where the diffusion process and deep CNN are implemented in a single step. We use two different methods for the end-to-end solution. In the first method, we use an alpha trainable network that computes the smoothness of the diffusion parameter (alpha), and the diffused image is then created using this computed alpha. This diffused image is then fed to a pre-trained three-layer CNN model (CNN layers with Batch Normalization) that gave 97.50% accuracy on the Replay-Attack dataset, and 99.62% accuracy on the Replay-Mobile dataset, respectively. In the second method, we fix a value for the smoothness of the diffusion parameter (alpha) using what we create for the diffused image, and then feed the diffused image to an Inception v4 network. In either case, we feed the original captured images to the framework, where the first layer computes the nonlinear diffusion based on an Additive Operator Splitting (AOS) scheme and an efficient block-solver called a Tri-Diagonal Matrix Algorithm (TDMA). This enhances the edges and preserves the boundary locations of the real image (similar to the work proposed in References [3,4]). This diffused input image is then fed to the deep CNN architecture or the Inception v4 network to extract the complex and deep features, and to classify the image as real or fake. Our integrated implementation results in real-time detection of liveness. We also do a comparison of our proposed integrated method with the previous approaches that have been proposed for liveness detection to determine how well it performs with regard to current state-of-the-art methods.

For the liveness detection on video frames, we propose a solution where we also first apply the nonlinear diffusion based on the AOS scheme and TDMA to the individual frames of the video sequence. This enhances the edges and preserves the boundary locations of the real image (similar to the static method proposed in References [3,4]). These diffused input images are then fed one-by-one to the CNN, which acts as the front-end of our architecture, and extracts the complex and deep features. The output of the CNN is then fed to the LSTM, which detects the temporal information in the sequence, and, finally, the output dense neural network layer classifies the sequence as real or fake. For the sake of completeness, we provide a brief summary of the key concepts in nonlinear diffusion.

### 3.1. Nonlinear Diffusion

Linear diffusion smoothens the input image at a constant rate in all directions to remove noise. Therefore, the smoothing process does not consider information regarding important image features such as edges [27]. The solution of the linear diffusion equation is given by:

(1) ∂I/∂t=div(d∇I),

where *I* is the image, *d* is the scalar diffusivity, and *div* is the divergence operator. This is somewhat equivalent to convolving the image with a Gaussian kernel, and, hence, linear diffusion can be regarded as a low pass filtering process.

The edge-preserving capability of nonlinear diffusion makes it a powerful denoising technique, as the information contained in high spatial frequency components is preserved [28]. Anisotropic diffusion, which is nonlinear diffusion based on a partial differential equation, prevents the blurring and localization issues associated with linear diffusion, and focuses on reducing the image noise without reducing significant parts of the image content such as edges. It improves the scale-space technique, enhances the boundaries, and preserves the edges [29]. The diffusion coefficient is locally adapted, and is chosen as a function of the image gradient, that varies with both the edge location and its orientation in order to preserve the edges. The nonlinear diffusion process is defined by the equation.

(2) ∂I/∂t=div(g(|∇I|)∇I),

where ∇I is the gradient, and the diffusivity g is a function of the gradient ∇I.

The Additive Operator Splitting (AOS) scheme addresses the problem of regularization associated with anisotropic diffusion [30]. This semi-implicit scheme is stable for all time-steps, and ensures that all co-ordinate axes are treated equally, as defined by Equation (3) [31]. AOS enables fast diffusion, resulting in smoothing of the edges in fake images while the edges in real images will be preserved. The iterative solution in AOS is given in Equation (3).

(3) $${\left(I_{k}\right)}^{t+1}=\overset{m}{\underset{l=1}{\sum }}{\left(mI-\tau m^{2}A_{l}\right)}^{-1}I_{k}^{t},$$

where *I_k_* is the diffused image, *m* is the number of dimensions, *k* represents the channel, *I* is the identity matrix, $A_{l}$. is the diffusion, and *τ* is the time steps (referred to as param. alpha in our implementation). In the two-dimensional case, *m* = 2, and the equation then becomes:

(4) $${\left(I_{k}\right)}^{t+1}={\left(2I-4\tau A_{1}\right)}^{-1}I_{k}^{t}+{\left(2I-4\tau A_{2}\right)}^{-1}I_{k}^{t},$$

where $A_{1}$ and $A_{2}$ denote the diffusion in the horizontal and vertical directions. The equation is split into two parts in the operator splitting scheme. The solution to each is computed separately and results are then combined.

The block-solver Tri-diagonal matrix algorithm (TDMA) is a simplified form of Gaussian elimination, useful in solving tri-diagonal systems of equations. The AOS scheme, together with TDMA can, therefore, be used to efficiently solve the nonlinear, scalar-valued diffusion equation [31]. We implement the AOS scheme in the first layer of our implementations.

### 3.2. End-to-End Diffusion-CNN Networks

#### 3.2.1. Specialized Convolutional Neural Network (SCNN)

We implemented a specialized end-to-end diffusion-CNN network (with Batch Normalization) where the smoothness of the diffusion parameter (alpha) is learned by the network. Convolutional Neural Networks (CNNs) work by combining the architectural concepts of local receptive fields, shared weights, and spatial or temporal subsampling in order to ensure some degree of shift, scale, and distortion invariance. To achieve higher accuracy, we use transfer learning by first training the CNN network on diffused images created with a fixed smoothness of diffusion value of 15. We then initialize the pre-trained convolutional neural network with batch normalization in the integrated diffusion architecture and retrain it again to obtain higher accuracy.

In our architecture, the original image is first fed to an alpha network to compute the value of the smoothness of diffusion parameter (alpha). This is a neural network comprising of a hidden layer of 15 neurons followed by a dense layer of one neuron, which outputs the alpha. The Rectified Linear Unit (ReLU) activation function is applied to the neurons in these layers. The SCNN model consists of three convolutional layers C1, C2, and C3 with 16, 32, and 64 feature maps, respectively, where kernel sizes of 15 × 15, 7 × 7, and 5 × 5 are used in the convolutions. Each convolution is followed by batch normalization, and max pooling is applied to the C1 and C2 layers after batch normalization for reducing the resolution. The higher filter size in the C1 layer is important to extract the diffusion enhanced features for liveness detection. The C3 layer batch normalization is followed by a dense layer of 64 neurons, and a dense output layer of one neuron. The ReLU activation function is applied to the convolution layers, the hidden layer, and the sigmoid activation function is applied to the output layer. The SCNN is trained using the binary-cross-entropy loss function, and the Adam optimizer with an initial learning rate set to 0.001.

**Figure 1:**
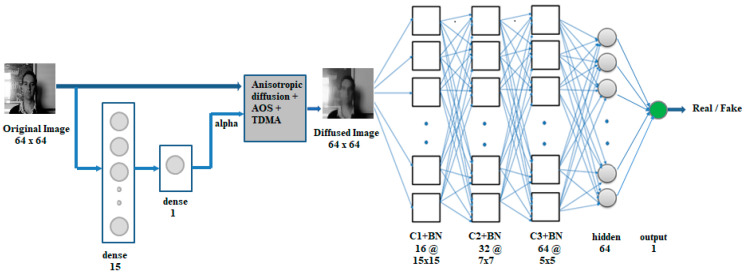
End-to-end architecture using the Specialized Convolutional Neural Network (SCNN) (alpha is learned by the network).

Figure 1 below shows the proposed architecture. The nonlinear diffusion code implemented in TensorFlow is used to convert the original image to diffused form with the parameter Alpha determined from the alpha network (bottom left in Figure 1). During backpropagation, the weights in the alpha network will be updated, and the updated weights are used in the computation of the output of the single neuron in the dense layer (parameter alpha) on the next forward pass.

#### 3.2.2. Inception v4

To determine if a more advanced CNN network will result in better accuracy, we replace the CNN part in Figure 1 with the Inception v4 network. However, instead of using an alpha network that computes the smoothness of diffusion (alpha), we fix the value of alpha in order to improve the real-time performance. The first stage is the nonlinear diffusion stage, whose input is the original 64 × 64 input image captured through the webcam, which is followed by the Inception network v4 architecture. The inception network is a CNN architecture designed as a deeper and wider network. It consists of inception modules stacked upon each other with intermittent subsampling layers for reducing the resolution and, thereby, reducing the shift and distortion in the image.

**Figure 2:**
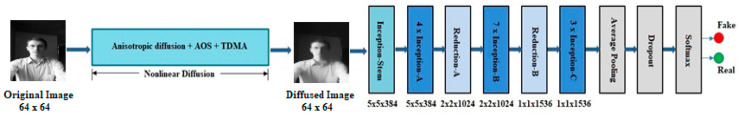
End-to-end architecture using the Inception v4 network.

The Inception network v4 architecture consists of an inception stem, three different inception blocks, namely inception-A, inception-B, and inception-C, which are used repeatedly 4, 7, and 3 times, respectively, and two reduction blocks for changing the resolution of the grid [4,32]. Convolutions in the inception modules within a block are performed by applying filters of multiple sizes to the same layer, making the network wider and enhancing the recognition of features at different scales. The resulting feature maps are then aggregated and forwarded to the next layer [33]. The complete architecture for this approach is illustrated in Figure 2. The diffused image obtained from the nonlinear diffusion stage using the fixed value of alpha is fed to the next stage, which is the inception stem of the Inception v4 network in which the output is then fed to the inception-A blocks, reduction-A block, Inception-B blocks, reduction-B block, and inception-C blocks, which is followed by an average pooling layer, dropout, and a dense output layer of two neurons with SoftMax activation. The network was trained by using the Adam optimization algorithm. Since our targets are in a categorical format with two classes of fake and real, we used the categorical cross-entropy as a loss function. The diffusion block was implemented via direct implementation of the diffusion equations, as described in Section 3.1.

### 3.3. CNN-LSTM

**Figure 3:**
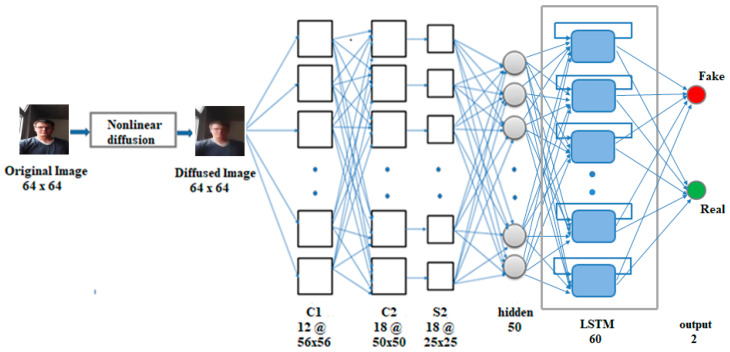
Convolutional Neural Network and Long Short-Term Memory (CNN-LSTM).

For liveness detection on video frames, we need to keep track of the information in a sequence of frames. Thus, our architecture for this case consists of diffusion, which is followed by CNN feeding to an LSTM layer, as shown in Figure 3. Multiplicative units called gates (input, output, forget) in each LSTM cell provide continuous analogues of write, read, and reset operations for the cells [34]. These units learn to open and close access to the constant error flow through internal states of the cells [35]. The input gate indicates how much of the new information must be stored in the cell state, the forget gate indicates how much of the internal state can be removed, and the output gate indicates how much of the cell state can be sent as output to the next time-step. LSTMs in combination with CNNs have been used successfully in person identification from lip texture analysis [36], 3D gait recognition [37], image-to-video person re-identification [38], and a deep bi-directional LSTM was used with CNN for action recognition in video sequences in Reference [39]. Our enhancement is the addition of the diffusion preprocessing to further enhance liveness detection.

The CNN part in our CNN-LSTM network has convolutional layers C1 and C2, one subsampling layer, and a fully connected layer of 50 neurons (adapted from the CNN-5 in Reference [4]). This is followed by the LSTM layer, which consists of 60 cells, and a feedforward output layer of two neurons. The sigmoid activation function is applied to the output layer, giving an output in the range 0 to 1. Nonlinear diffusion is first applied to the frames in each input sequence, and the diffused frames are fed to the CNN. The CNN captures the spatial information in the sequence by extracting the complex and deep discriminative features, and the hidden layer of CNN produces an output of 50 features per frame. The input to the LSTM layer is three-dimensional, where the three dimensions are samples, time-steps, and features (i.e., batch size, 20, 50), where 20 is the number of frames (time-steps) per sequence. The LSTM layer captures the long-term temporal dependencies across frames in the sequence, and the 60 features obtained from the LSTM layer are fed to the output layer, which then classifies the 20-frame sequence as real or fake. The network is trained by backpropagation through time using the Adam optimization algorithm with mean-squared-error as the loss function, and batch size set to 32. Implementation of the diffusion block was done via direct implementation of the diffusion equations.

## 4. Performance Evaluation

We performed experimental evaluations using our proposed end-to-end diffusion–SCNN architecture, and the end-to-end diffusion-Inception v4 architecture on the Replay-Attack dataset and the Replay-Mobile dataset. We describe the experimental results, and the hyper-parameter settings used in the experiments. We also do a performance comparison with other existing liveness detection methods on static images. For liveness detection on video sequences, we performed experimental evaluations using the CNN-LSTM architecture on the Replay-Attack dataset and Replay-Mobile dataset. We present the experimental results, and the hyper-parameter settings used in the experiments. We further do a performance comparison with other existing liveness detection methods on video sequences.

### 4.1. Datasets

#### 4.1.1. Replay-Attack Dataset

The Replay-Attack database [22] is a 2D face spoofing attack database that consists of 1200 short video recordings of real-access and attack attempts of 50 different subjects. The frames in the video clip have a resolution of 320 × 240 pixels. As shown in Table 1, the data is divided into three sub-groups comprising of training, development, and test sets. Clients that appear in one dataset do not appear in any other dataset. Both real and attack videos were taken under two different lighting conditions including controlled and adverse. There are 4 real and 20 attack videos per subject. The attack videos include four mobile attacks using an iPhone screen, four screen attacks using an iPad screen, and two hard-copy print attacks with each captured in two different modes, hand-based attacks, and fixed-support attacks. The training set consists of 15 subjects and 360 video clips. The development set consists of 15 subjects and 360 video clips, and the testing set consists of 20 subjects and 480 video clips.

#### 4.1.2. Replay-Mobile Dataset

The Replay-Mobile dataset [20] consists of 1030 video clips of photo and video attacks of 40 clients. The videos were recorded under different lighting conditions with an iPad Mini2 (running iOS) and an LG-G4 smartphone (running Android), and the frames in the video clips are of a 720 × 1080 resolution. The videos are grouped into training set, development, and test set. These sets are disjoint, and, therefore, clients in one set do not appear in the other sets. The real-access videos were taken under five different lighting conditions (controlled, adverse, direct, lateral, diffuse). In order to produce the attacks, high-resolution photos and videos from each client were used under similar conditions as in their authentication sessions (light on and light off).

For the real-access, each client recorded 10 videos, with two videos in each of the five lighting conditions. Two kinds of attacks were performed including matte-screen attacks and print attacks. Each client recorded 16 attack videos, which include four mobile attacks and four tablet attacks using a mattescreen. Two print attacks were each captured by fixed-support and hand-held smartphone. Two print attacks were each captured by fixed-support and a hand-held tablet. There are 12 subjects in the training set with 120 real-access and 192 attack videos, 16 subjects in the development set with 160 real-access and 256 attack videos, and 12 subjects in the test set with 110 real-access videos (since one subject was not available) and 192 attack videos (Table 2).

### 4.2. Experimental Setup

Our experiments were implemented on an Intel Xeon E3-1271 @3.60GHz with 32GB RAM PC. For diffusing the captured image in the end-to-end real-time liveness detection of a static image, we implemented the diffusion equations, as described in Section 3.1 to integrate with the TensorFlow. The SCNN and Inception v4 code were also written using TensorFlow. The complete code implements the framework by doing the implementation and classification in a single step. For the CNN-LSTM, the diffusion equations were implemented using the Matlab code (version 9.8, R2020a) by the author of Reference [31], and the implementation of the CNN-LSTM was carried out in TensorFlow.

### 4.3. Experimental Results

#### 4.3.1. End-to-End Diffusion—CNN Networks

**Figure 4:**
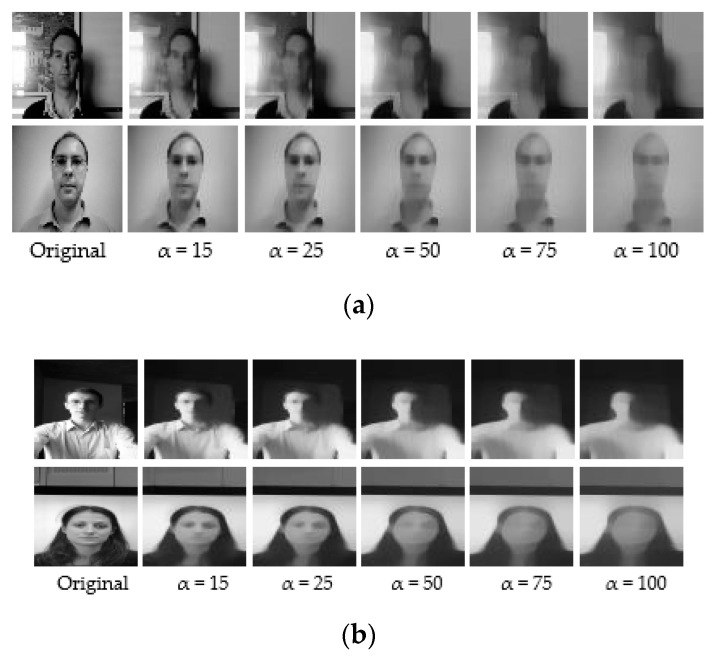
Sample images from the datasets and their corresponding diffused versions. (**a**) Images from the Replay-Attack dataset. (**b**) Images from the Replay-Mobile dataset. The images in the first row of both (**a**,**b**) are real, and the images in the second row of both (**a**,**b**) are fake.

For the end-to-end networks, we conducted experiments on the Replay-Attack dataset and the Replay-Mobile dataset. The original images read from folders were grayscaled and then fed to the end-to-end architectures. Figure 4 shows some samples from the Replay-Attack dataset and the Replay-Mobile dataset, and the corresponding diffused images. The first row in both (a) and (b) shows real images from the Replay-Attack and Replay-Mobile datasets, respectively, and the second row in (a) and (b) shows fake images. The first column in (a) and (b) are original non-diffused images, and the remaining columns are their diffused versions created with a parameter alpha set to 15, 25, 50, 75, and 100, respectively. It can be observed that a value below 50 produces better diffused images with highlighted edges and enhanced boundaries in the real image, whereas a high value of 75/100 for the smoothness parameter alpha blurs out important edges from the images.

We conducted numerous experiments individually on the grayscaled images of the Replay-Attack dataset and the Replay-Mobile dataset. We tuned the hyperparameters by validating on the validation set during training, and the best model obtained was evaluated on the test set. We computed the test accuracy, and the Half Total Error Rate (HTER), which is defined as:

HTER = (FAR + FRR)/2,(5)

where FAR is the False Acceptance Rate, and FRR is the False Rejection Rate.

FAR = False Acceptance/Number of Impostor images(6)

FRR = False Rejection/Number of Real images(7)

We used 20 frames of each video clip in the training, development, and testing sets, which are resized to a size of 64 × 64. Thus, a total of 7200 training images, 7200 developmental set (validation) images, and 9600 testing images were used from the Replay-Attack dataset, and 6240 training images, 8320 development set images, and 6040 test set images were used from the Replay-Mobile dataset.

##### SCNN

Using the Specialized Convolutional Neural Network (SCNN), our experiments on the Replay-Attack dataset gave test accuracy of 96.03% and hter of 7.53%, and 96.21% test accuracy and 4.96% hter on the Replay-Mobile dataset. We first trained the three-layer CNN on diffused training images created with an alpha parameter of 15 for 100 epochs by validating on the diffused validation images created with the same alpha, for tuning the hyperparameters. We experimented with various learning rates, and the best model obtained gave an accuracy of 97.50% at a learning rate of 0.005 for the Replay-Attack dataset, and 99.62% accuracy at a learning rate of 0.0005 for the Replay-Mobile dataset. We used these pre-trained models in the CNN part of the end-to-end SCNN architecture. We trained the SCNN network on the original training images for 30 epochs while validating on the original images of the validation set. We used the Adam optimizer, binary-cross-entropy loss function, and sigmoid activation function in the output layer. The training was done by setting the learning rate of the optimizer to its default value (0.001), and also using the learning rates, which gave the best model of the pre-trained CNN-3, which were 0.005 for the Replay-Attack dataset, and 0.0005 for the Replay-Mobile dataset. The smoothness of diffusion (alpha) was learned by the network each time. The original image fed to the network was diffused with this learned alpha, and the diffused image was then fed to the pre-trained 3-layer CNN. The weights of the better model were saved during the 30 epochs of training. Following training, the best model weights obtained during validation were loaded, and the network model was compiled and evaluated on the test set. The experimental results are summarized in Table 3 below.

The real-time evaluation of a test image on a trained SCNN network takes approximately 0.021 s for the Replay-Attack dataset, and 0.016 s for the Replay-Mobile dataset.

##### Inception v4 Network

For the end-to-end solution using Inception v4 network (Figure 2), we trained the network on the original images of the training set for 30 epochs using a fixed value for the smoothness of the diffusion parameter (parameter alpha) while validating on the original images of the validation (development) set for tuning the hyperparameters and selecting the best model for evaluation. We repeated the training process for various values of alpha and learning rates of the optimizer used. The best model obtained was then evaluated on the test set. We used the Adam optimizer, categorical cross-entropy loss function, and SoftMax classifier in the last stage with a batch size set to 32. The tables and plots below summarize the results obtained.

- Results Obtained with the Replay-Attack Dataset

**Figure 5:**
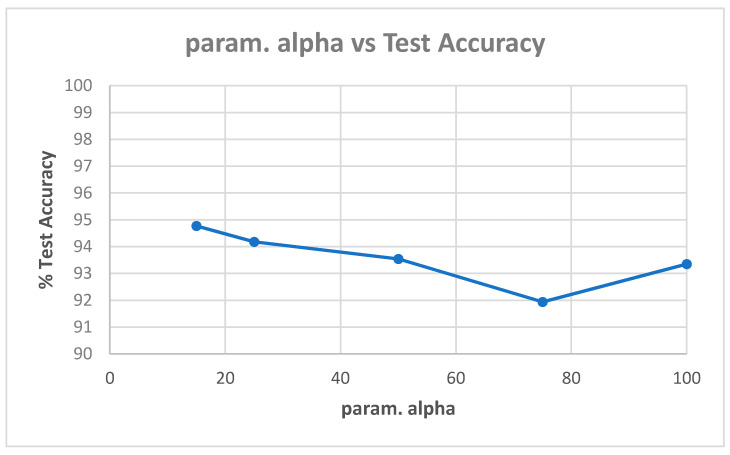
Plot showing parameter alpha vs. test accuracy (Table 4).

**Figure 6:**
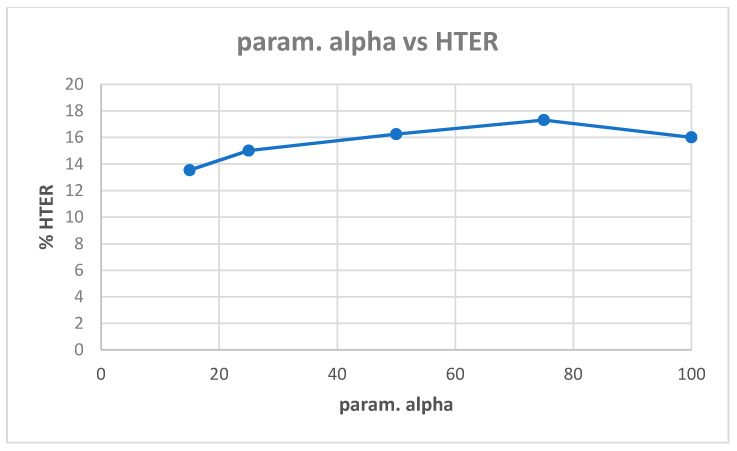
Plot showing parameter alpha vs. HTER (Table 4).

Table A1 in Appendix A shows the best model (highest validation accuracy) results obtained for various values of alpha with the Replay-Attack dataset when validating on the validation set during training for 30 epochs for tuning the hyperparameters. The overall best model obtained was for alpha of 15, at the default learning rate of 0.001. The results of evaluation of the best models for each alpha in Table A1, on the test set of the Replay-Attack dataset, are shown in Table 4. It can be observed that the best results obtained are test accuracy of 94.77%, and HTER of 13.54% for alpha of 15. The plots of alpha vs. test accuracy and alpha vs. HTER, according to results in Table 4, are shown in Figure 5 and Figure 6.

On a trained Inception v4 network, real-time evaluation of a test image takes approximately 0.016 s for the Replay-Attack dataset.

- Results Obtained with the Replay-Mobile Dataset

**Figure 7:**
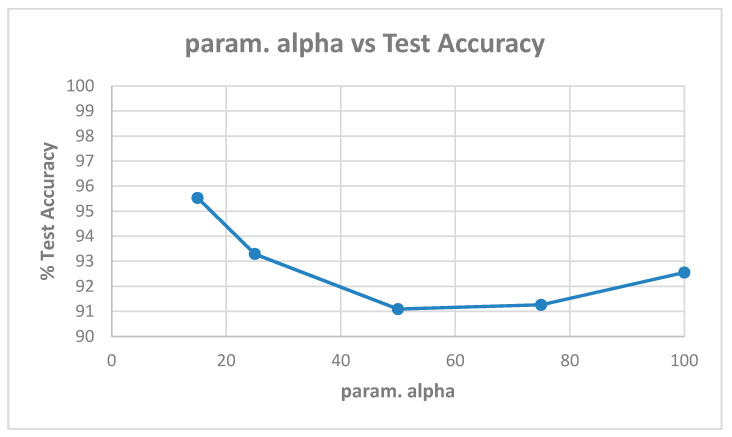
Plot showing parameter alpha vs. test accuracy (Table 5).

**Figure 8:**
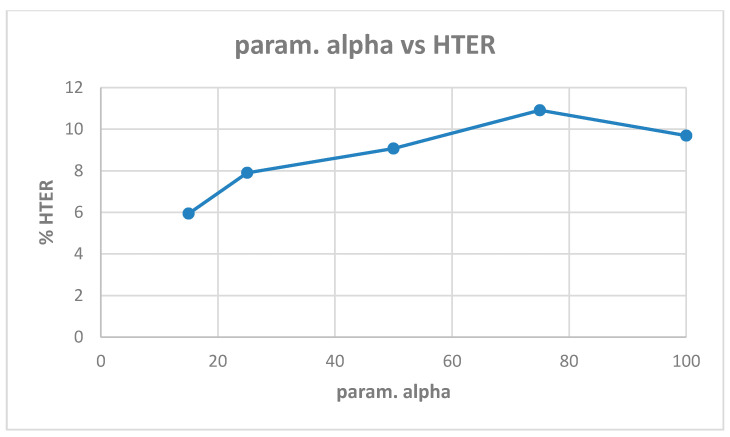
Plot showing parameter alpha vs. HTER (Table 5).

Table A2 shows the best model (highest validation accuracy) results obtained for various values of alpha with the Replay-Mobile dataset when validating on the validation set during training for 30 epochs for tuning the hyperparameters. The overall best model obtained was for alpha of 15, at the default learning rate of 0.001. The results from evaluating the best models for each alpha (Table A2) on the test set of the Replay-Mobile dataset are shown in Table 5. The best results obtained are test accuracy of 95.53%, and HTER of 5.94% for alpha of 15. The plots of alpha vs. test accuracy and alpha vs. HTER, according to results in Table 5, are shown in Figure 7 and Figure 8.

On a trained Inception v4 network, real-time evaluation of a test image takes approximately 0.019 s for the Replay-Mobile dataset.

##### Comparison with State-of-the-Art Methods

**Figure 9:**
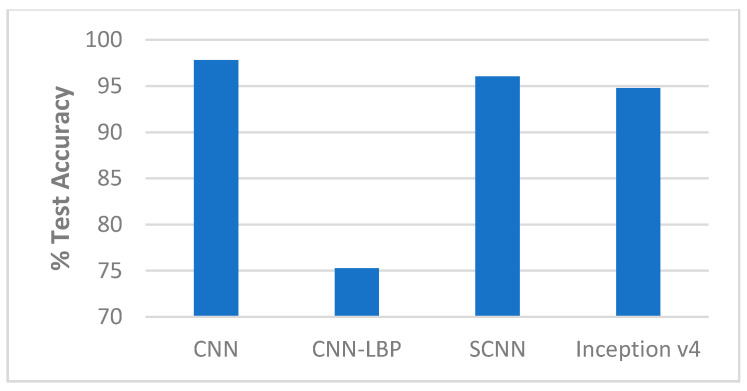
Performance comparison (% Test accuracy) of the end-to-end networks on the Replay-Attack dataset (Table 6).

**Figure 10:**
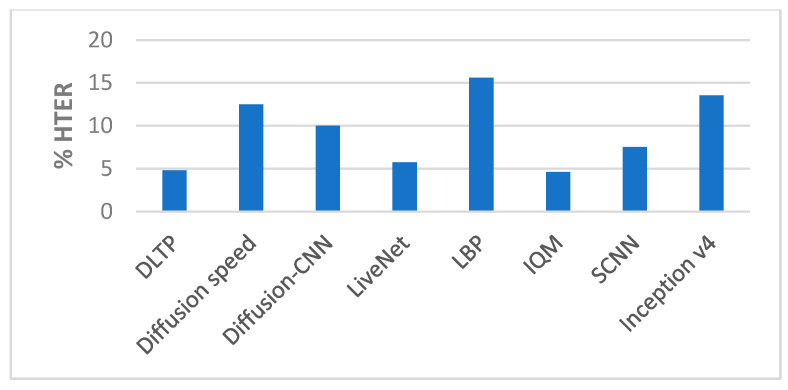
Performance comparison (% HTER) of the end-to-end networks on the Replay-Attack dataset (Table 6).

**Figure 11:**
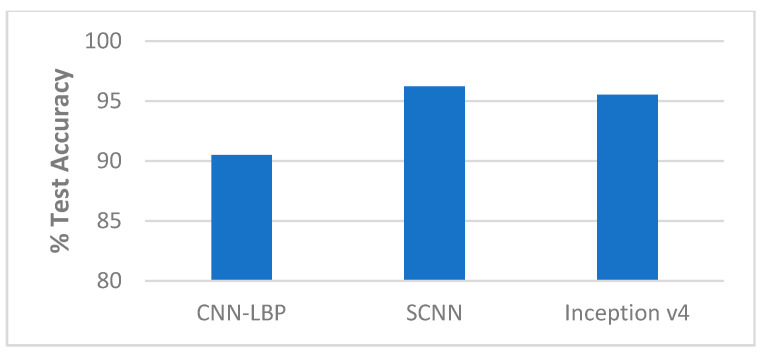
Performance comparison (% Test accuracy) of the end-to-end networks on the Replay-Mobile dataset (Table 7).

**Figure 12:**
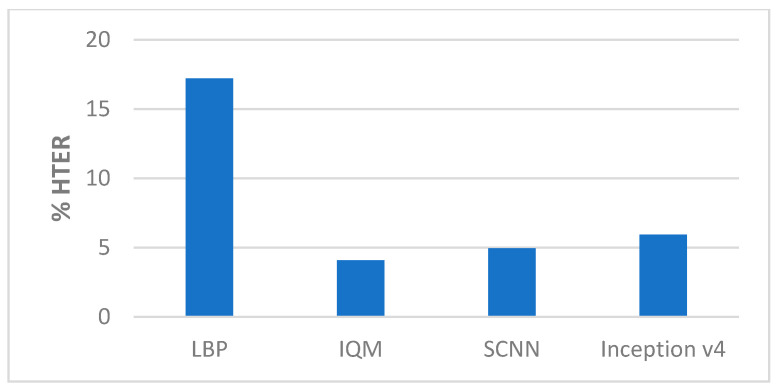
Performance comparison (% HTER) of the end-to-end networks on the Replay-Mobile dataset (Table 7).

The performance of our end-to-end approaches using the SCNN and Inception v4 were compared with other proposed methods on the Replay-Attack dataset and Replay-Mobile dataset, as shown in Table 6 and Table 7 and Figure 9, Figure 10, Figure 11 and Figure 12.

Unlike the other methods, our proposed method is an end-to-end solution capable of liveness detection in real-time. Even though the accuracy and HTER are slightly below the best reported model, the tradeoff of real-time performance is an important goal. Therefore, these architectures are capable of providing an end-to-end solution with the advantage of being real-time for use in face recognition applications.

#### 4.3.2. CNN-LSTM

**Figure 13:**
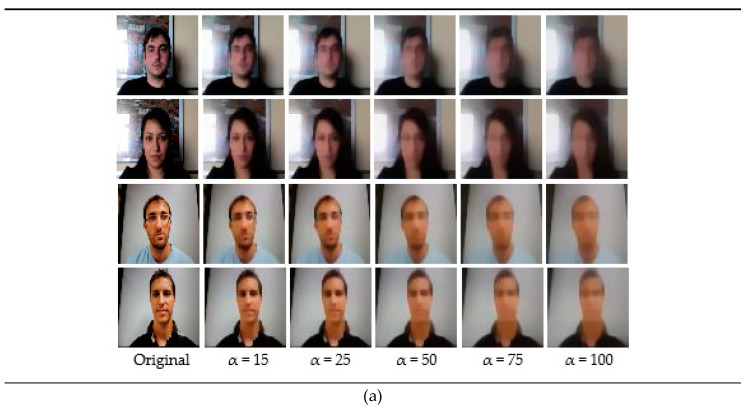

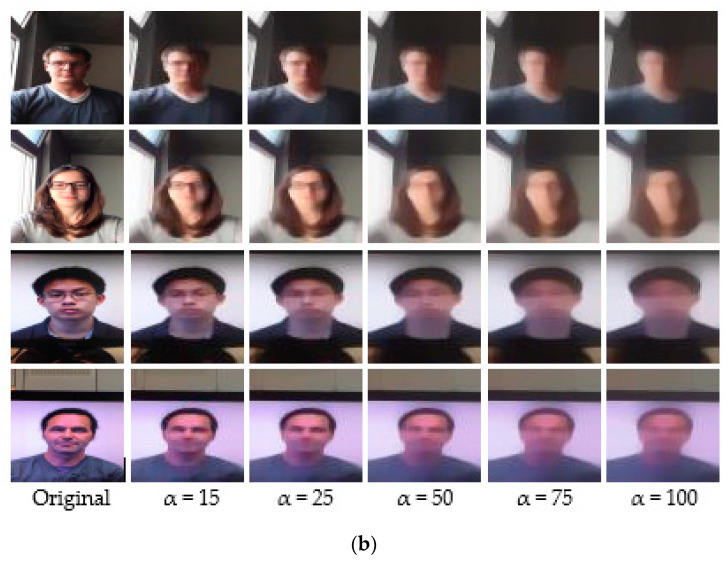
Sample images from the datasets and their corresponding diffused versions. (**a**) Images from the Replay-Attack dataset. (**b**) Images from the Replay-Mobile dataset. The images in the first two rows of both (**a**,**b**) are real, and the images in the third and fourth row of both (**a**,**b**) are fake.

For liveness detection on videos, we used 20 frames of each video clip in the training, development, and test sets. We resized the frames to a size of 64 × 64, and created five sets of diffused images for our experiments with different values (15, 25, 50, 75, and 100) of the parameter param. alpha that defines the smoothness of diffusion. Figure 13 below shows some samples from the Replay-Attack and Replay-Mobile dataset, and their corresponding diffused versions. The first two rows in both (a) and (b) show real images from the Replay-Attack and Replay-Mobile datasets, respectively, and the second and third rows in (a) and (b) show fake images in the datasets. The images in the first column in (a) and (b) are original non-diffused images, and the images in the remaining columns are their diffused versions created with the parameter alpha set to 15, 25, 50, 75, and 100, respectively. We tested our proposed framework with each of these sets of diffused images. We conducted numerous experiments on the Replay-Attack dataset and Replay-Mobile dataset by changing the hyper-parameters during the learning phase.

We computed the test accuracy of the CNN-LSTM architecture after training for various cases using diffused images created with param. alpha set to 15, 25, 50, 75, 100. We used the Adam optimizer and mean-squared-error loss function. The activation functions used were ReLU for the convolutional layers and hidden layer, and sigmoid for the output layer. The number of neurons in the hidden layer of the classifier was set to 50, and the number of cells in the LSTM was set to 60. We trained the CNN-LSTM network for 100 epochs on the training set, while validating on the validation (development) set for tuning the hyperparameters. During each epoch of training, if a better validation accuracy was obtained, the model was saved. We repeated this process for various values of learning rates. At the end of training, the saved model (i.e., the one that gave the highest validation accuracy) was loaded, and then evaluated on the test set for the test accuracy and HTER. We obtained 98.71% test accuracy and 2.77% HTER on the Replay-Attack dataset, and 95.41% test accuracy and 5.28% HTER on the Replay-Mobile dataset. The tables and plots below summarize our results.

##### Results Obtained with the Replay-Attack Dataset

**Figure 14:**
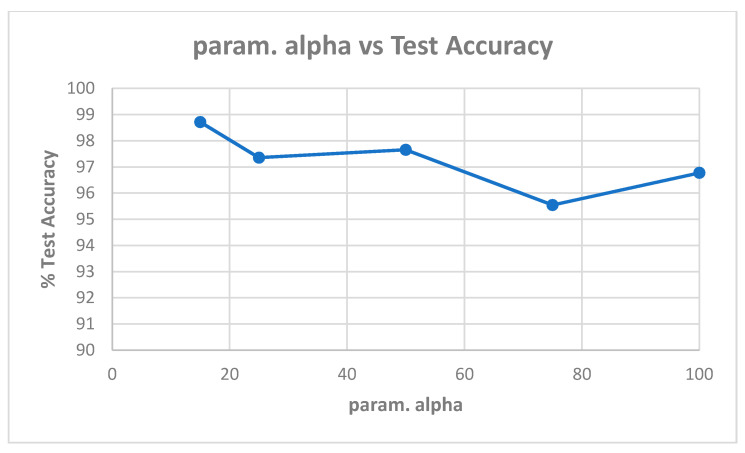
Plot showing parameter alpha vs. test accuracy (Table 8).

**Figure 15:**
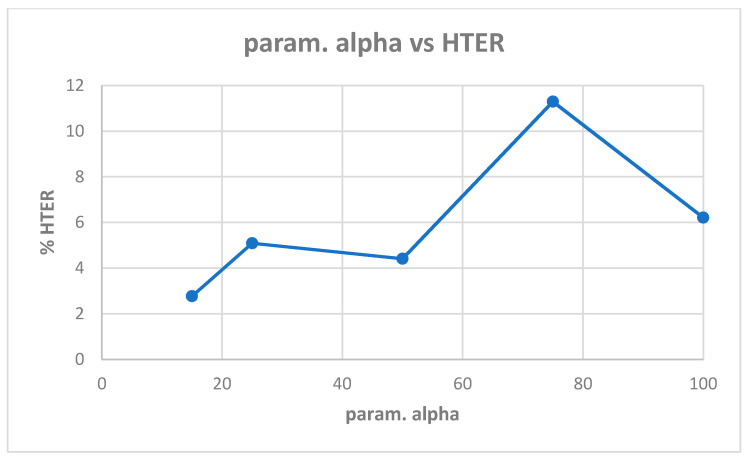
Plot showing parameter alpha vs. HTER (Table 8).

Table A3 shows the best model (highest validation accuracy) results obtained with the Replay-Attack dataset when validating on the validation set during training for 100 epochs for tuning the hyperparameters. The results of evaluation of the best models for each alpha in Table A3, on the test set of the Replay-Attack dataset, is shown in Table 8. It can be observed that the best results obtained are test accuracy of 98.71% and HTER of 2.77% with diffused images created with alpha of 15. The plots of alpha vs. test accuracy and alpha vs. HTER, according to the results in Table 8, are shown in Figure 14 and Figure 15.

We performed experiments without diffusion, i.e., by feeding the original non-diffused images directly to the network. The best validation accuracy obtained while validating on the validation set during training was 97.94%, as shown in Table A3. This model, when loaded and evaluated on the non-diffused original images of the test set, gave 96.27% test accuracy and 8.31% HTER (Table 8). Therefore, by applying diffusion, there is a significant improvement in accuracy and HTER by 2.44% and 5.54%, respectively.

**Figure A1:**
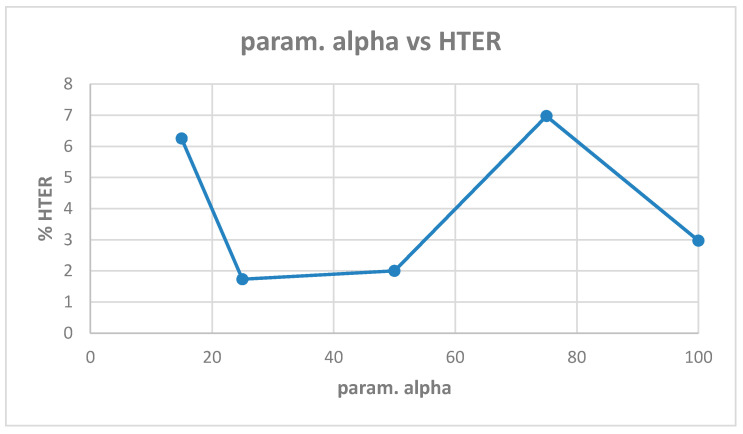
Plot showing parameter alpha vs. HTER (Table A5).

We also computed test results by training the network on the train set, and validating on the testing set. The Table A4 and Table A5, and Figure A1 show the results obtained.

##### Results Obtained with the Replay-Mobile Dataset

**Figure 16:**
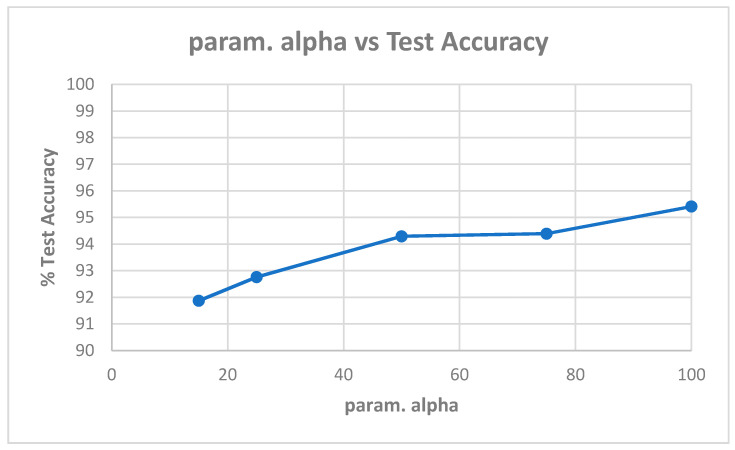
Plot showing parameter alpha vs. test accuracy (Table 9).

**Figure 17:**
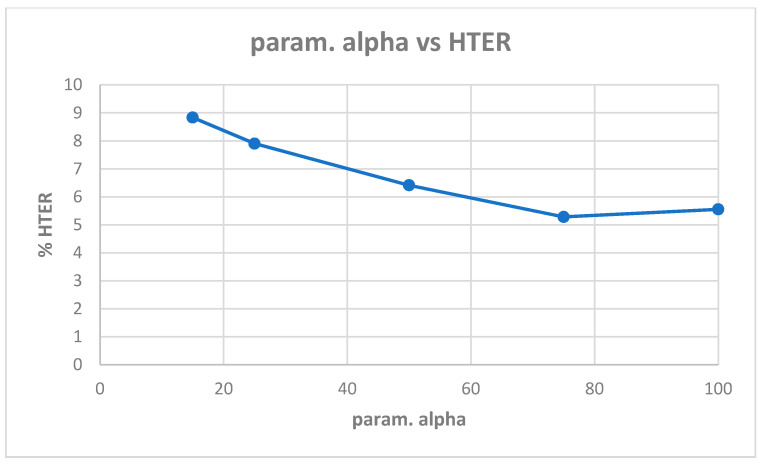
Plot showing parameter alpha vs. HTER (Table 9).

Table A6 shows the best model (highest validation accuracy) results obtained with the Replay-Mobile dataset when validating on the validation set during training for 100 epochs for tuning the hyperparameters. The results of evaluation of the best models for each alpha (Table A6) on the test set of the Replay-Mobile dataset, are shown in Table 9, and the corresponding plots are shown in Figure 16 and Figure 17. The best results obtained are the test accuracy of 95.41%, and HTER of 5.28% for alpha of 100 and 75.

In the experiments performed without diffusion, i.e., by feeding the original non-diffused images directly to the network, the best validation accuracy obtained while validating on the validation set during training was 98.97%, as shown in Table A6. This model, when loaded and evaluated on the original non-diffused test set images, gave 95.20% test accuracy and 5.91% HTER (Table 9). In this case, by applying diffusion, though there is no significant improvement as obtained with the Replay-Attack dataset, there is still a slight improvement in accuracy and HTER.

**Figure A2:**
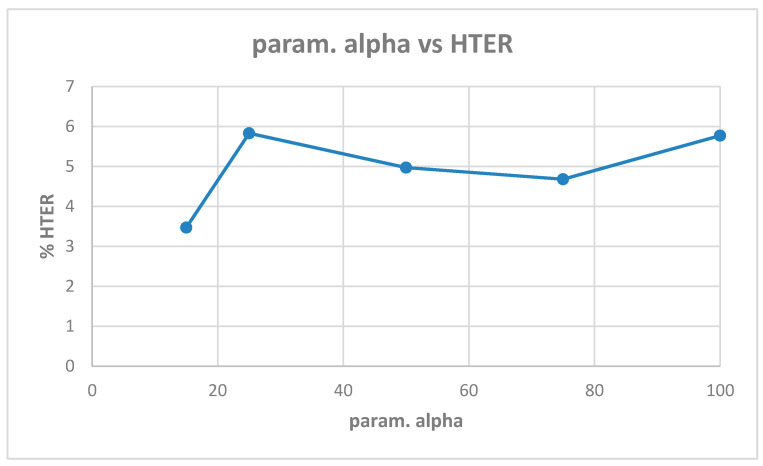
Plot showing parameter alpha vs. HTER (Table A8).

We also computed test results by training the network on the train set, and validating on the testing set. The Table A7 and Table A8, and Figure A2 show the results obtained.

**Figure 18:**
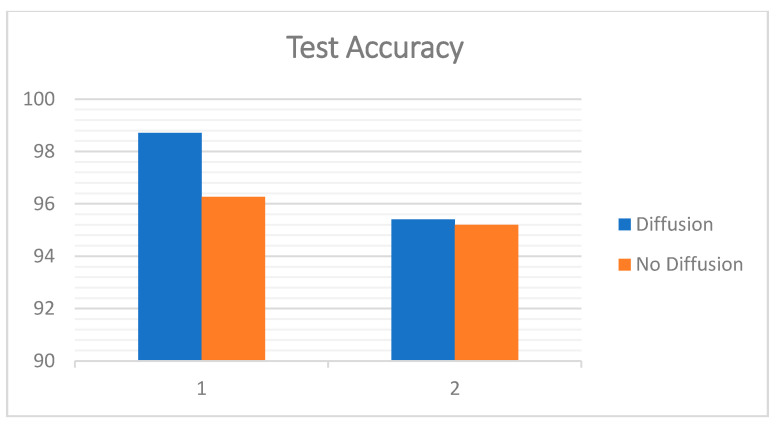
Test accuracy (%) obtained with and without diffusion for the Replay-Attack and Replay-Mobile datasets (1: Replay-Attack, 2: Replay-Mobile).

**Figure 19:**
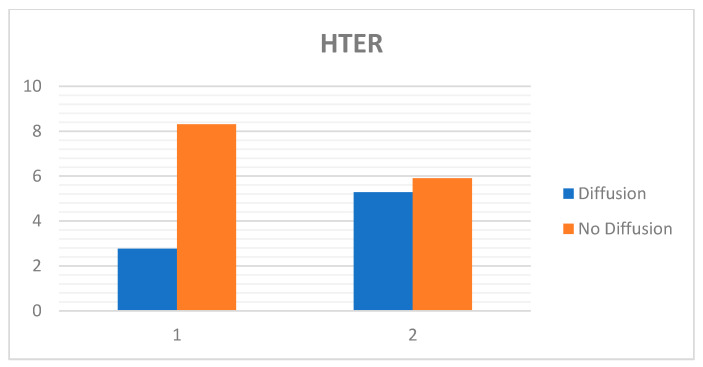
HTER (%) obtained with and without diffusion for Replay-Attack and Replay-Mobile datasets (1: Replay-Attack, 2: Replay-Mobile).

The charts below (Figure 18 and Figure 19) show the best results with diffusion and the results without diffusion for both the Replay-Attack dataset (Table 8) and the Replay-Mobile dataset (Table 9).

The training of the CNN-LSTM framework is very fast, as it takes only about 15 min for the Replay-Attack dataset, and 14 min for the Replay-Mobile dataset.

##### Comparison with State-of-the-Art Methods

**Figure 20:**
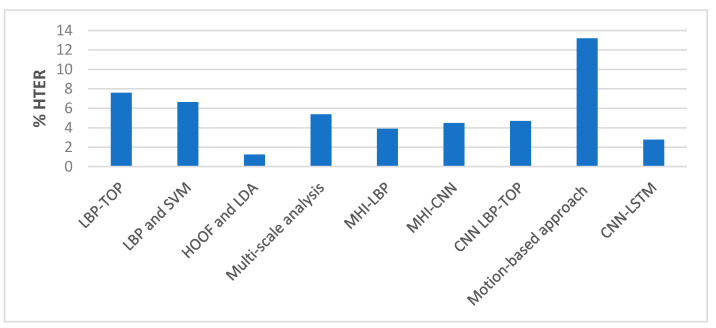
Performance comparison (HTER) on the Replay-Attack dataset (Table 10).

**Figure 21:**
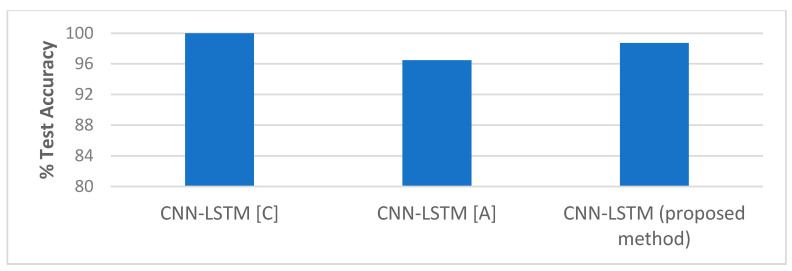
Performance comparison (Test Accuracy) on the Replay-Attack dataset (Table 10).

The performance of our proposed approach was compared with state-of-the-art methods for liveness detection on the Replay-Attack dataset, as shown in Table 10, and Figure 20 and Figure 21. In Reference [12], an HTER of 7.60% was achieved, and, in Reference [13], they achieved HTER of 6.62% using LBP and SVM, and HTER of 1.25% using HOOF and LDA on the Replay-Attack dataset. In Reference [15], 5.38% HTER was achieved using the multi-scale analysis, and, in Reference [16], the MHI-LBP gave HTER of 3.9% and MHI-CNN gave HTER of 4.5%. The CNN LBP-TOP method proposed in Reference [17] gave HTER of 4.7%. In the work proposed in Reference [19] that makes use of motion cues for face anti-spoofing while 100% and 96.47% accuracy were achieved when tested separately on Replay-Attack (controlled) and Replay-Attack (adverse) test sets. The work proposed in Reference [21] reported HTER of 13.2%. We achieved 98.71% accuracy and 2.77% HTER when we tested our proposed framework on the entire testing set of the Replay-Attack database.

In the above table, some of the entries are blank because the test accuracy and HTER were not reported by the authors.

**Figure 22:**
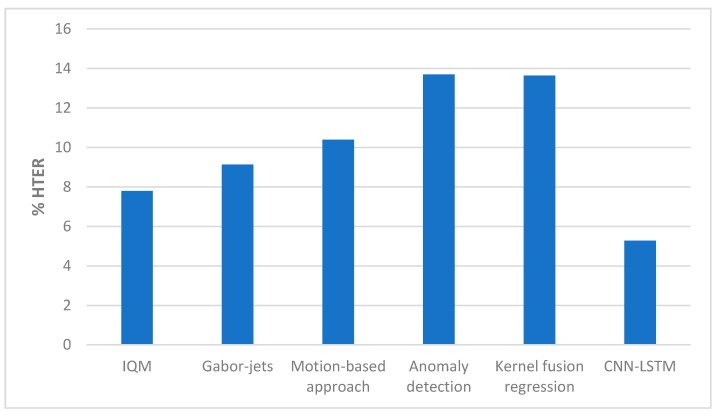
Performance comparison (HTER) on the Replay-Mobile dataset (Table 11).

Table 11 and Figure 22 below shows the comparison of our method with state-of-the-art methods on the Replay-Mobile dataset. In Reference [20], HTER of 7.80% was achieved for IQM, and HTER of 9.13% was achieved for Gabor-jets. In the work proposed in Reference [21], HTER of 10.4% was achieved. The anomaly detection approach proposed in Reference [25] gave HTER of 13.70%, and the one-class multiple kernel fusion regression approach proposed in Reference [26] gave 13.64% HTER.

As shown in Table 10 and Table 11, our architecture gave very competitive results when compared to other state-of-the-art methods in the literature for the Replay-Attack dataset, and gave the lowest HTER when compared to state-of-the-art methods for the Replay-Mobile dataset. This proves that it is an efficient solution for use in face recognition applications that require liveness detection on video frames for anti-spoofing.

## 5. Conclusions

We have developed face liveness detection architectures for static as well as video frames. For static images, our approach is an end-to-end real-time solution to the face liveness detection problem. Previous best approaches relied on a separate preprocessing step for creating diffused images whereas our work has integrated the diffusion process as well as the face liveness classification into a single TensorFlow application. We used a specialized CNN (SCNN) and the Inception v4 network in conjunction with the anisotropic diffusion for liveness classification. An analysis of the various test results obtained shows that the smoothness of the diffusion is an important factor in determining the livelihood of the captured image. We determined that the proposed framework gives better results with lower values of the smoothness parameter since higher values of this parameter blur out important information from the image. The nonlinear diffusion together with the CNN’s capability of recognizing features at different scales enhances the recognition rate. Our proposed framework for both the end-to-end solutions produced promising results on the Replay-Attack and Replay-Mobile datasets, compare favourably to other state-of-the-art methods in the literature, and it has the added advantage of accomplishing face liveness detection in real-time.

We also proposed a solution for face liveness detection in video sequences using a combination of diffusion (CNN and LSTM). We first applied nonlinear diffusion to each frame in the sequence, which makes the edge information and surface texture of a real image more pronounced than that of a fake image. The CNN extracts the complex and deep spatial features of each frame, and the LSTM captures the temporal dynamics in the sequence in order to classify the video sequence as real or fake. Our architecture produced competitive results compared to other state-of-the-art methods in the literature. Our experiments with the Replay-Attack dataset produced 98.71% test accuracy and an HTER of 2.77%, and our experiments on the Replay-Mobile dataset gave test accuracy of 95.41% and HTER of 5.28%, proving that it is a successful method for liveness detection of sequences. Our future work on the end-to-end solution will try to improve the classification accuracy by experimenting with various hyper-parameters, and then try the improved framework for detecting face spoofing attacks on video streams. As for the CNN-LSTM, our future work would be to improve the accuracy by experimenting with various hyper-parameters as well as deeper architectures.

## Figures and Tables

**Table 1:** Replay-Attack Dataset.

	Number of Subjects	Real-Access Videos	Attack Videos	Total
Training set	15	60	300	360
Development set	15	60	300	360
Testing set	20	80	400	480
Total	50	200	1000	1200

**Table 2:** Replay-mobile dataset.

	Number of Subjects	Real-Access Videos	Attack Videos	Total
Training set	12	120	192	312
Development set	16	160	256	416
Testing set	12	110	192	302
Total	40	390	640	1030

**Table 3:** Highest validation accuracy (best model) obtained during validation, and the test results obtained by evaluating the best model on the test set for the Specialized Convolutional Neural Network (SCNN).

	Replay-Attack	Replay-Mobile
Best model (%)	95.04	98.56
Test Accuracy (%)	96.03	96.21
HTER (%)	7.53	4.96

**Table 4:** Test results obtained with the Replay-Attack dataset by evaluating the best model obtained for each alpha shown in Table A1, on the test set, in Inception v4.

Alpha	15	25	50	75	100
Test accuracy (%)	94.77	94.18	93.54	91.94	93.35
HTER (%)	13.54	15.01	16.25	17.31	16.01

**Table 5:** Test results obtained with the Replay-Mobile dataset by evaluating the best model obtained for each alpha in Table A2, on the test set, in Inception v4.

Alpha	15	25	50	75	100
Test accuracy (%)	95.53	93.29	91.09	91.26	92.55
HTER (%)	5.94	7.90	9.07	10.91	9.69

**Table 6:** Comparison with state-of-the-art methods on the Replay-Attack dataset (the results of our proposed methods are highlighted in bold).

Method	Test Accuracy	HTER
DLTP [1]		4.8%
Diffusion speed [2]		12.50%
Diffusion-CNN [3]		10%
LiveNet [7]		5.74%
CNN [5]	97.83%	
CNN-LBP [6]	75.25%	
LBP [21]		15.6%
IQM [21]		4.6%
SCNN (proposed method)	**96.03%**	**7.53%**
Inception v4 (proposed method)	**94.77%**	**13.54%**

**Table 7:** Comparison with state-of-the-art methods on the Replay-Mobile dataset (the results of our proposed methods are highlighted in bold).

Method	Test Accuracy	HTER
CNN-LBP [6]	90.52%	
LBP [21]		17.2%
IQM [21]		4.1%
SCNN (proposed method)	**96.21%**	**4.96%**
Inception v4 (proposed method)	**95.53%**	**5.94%**

**Table 8:** Test accuracies and HTER obtained by evaluating the best models of each alpha (highlighted in Table A3) on the test set (the highest test accuracy and lowest HTER are indicated in bold).

Alpha	15	25	50	75	100	Without Diffusion
Test accuracy (%)	**98.71**	97.35	97.65	95.54	96.77	96.27
HTER (%)	**2.77**	5.09	4.41	11.29	6.21	8.31

**Table 9:** Test accuracies and HTER obtained by evaluating the best models of each alpha (highlighted in Table A6) on the test set (the highest test accuracy and lowest HTER are indicated in bold).

Alpha	15	25	50	75	100	Without Diffusion
Test accuracy (%)	91.87	92.76	94.29	94.39	**95.41**	95.20
HTER (%)	8.33	7.90	6.41	**5.28**	5.55	5.91

**Table 10:** Comparison with state-of-the-art methods on the Replay-Attack dataset.

Method	Test Accuracy	HTER
LBP-TOP [12]		7.60%
LBP and SVM [13]		6.62%
HOOF and LDA [13]		1.25%
Multi-scale analysis [15]		5.38%
MHI-LBP [16]		3.9%
MHI-CNN [16]		4.5%
CNN LBP-TOP [17]		4.7%
CNN-LSTM (C) [19]	100%	
CNN-LSTM (A) [19]	96.47%	
Motion-based approach [21]		13.2%
CNN-LSTM (proposed method)	98.71%	2.77%

**Table 11:** Comparison with state-of-the-art methods on the Replay-Mobile dataset.

Method	Test Accuracy	HTER
IQM [20]		7.80%
Gabor-jets [20]		9.13%
Motion-based approach [21]		10.4%
Anomaly detection [25]		13.70%
Kernel fusion regression [26]		13.64%
CNN-LSTM (proposed method)	95.41%	5.28%

## Appendix A

**Table A1:** Best model obtained for each alpha while validating on the development set during training, with the Replay-Attack dataset, in Inception v4.

Alpha	15	25	50	75	100
Val. Acc.	94.81	94.25	93.40	93.44	93.31

**Table A2:** Best model obtained for each alpha while validating on the development set during training, with the Replay-Mobile dataset, in Inception v4.

Alpha	15	25	50	75	100
Val. Acc. (%)	98.20	96.98	96.49	95.43	94.66

**Table A3:** Highest validation accuracy (best model) obtained for different values of learning rate, by validating on diffused validation images created with each alpha, during training, with Replay-Attack dataset, in CNN-LSTM (the highest validation accuracy for each alpha, and without diffusion, is highlighted in bold).

Alpha	Learning Rate				
	0.001	0.002	0.005	0.0008	0.0005
15	98.22	**98.90**	95.24	97.46	97.18
25	**99.13**	95.56	94.74	98.89	97.78
50	96.00	**98.58**	96.71	98.07	97.51
75	**97.56**	96.11	96.53	96.17	97.50
100	96.94	95.07	95.53	**98.61**	98.33
w/o diffusion	97.28	95.46	96.67	**97.94**	97.54

**Table A4:** Highest validation accuracy (best model) obtained for different values of learning rate, by validating on diffused test images created with each alpha, during training, with Replay-Attack dataset, in CNN-LSTM (the highest validation accuracy for each alpha, and without diffusion, is highlighted in bold).

Alpha	Learning Rate				
	0.001	0.002	0.005	0.0008	0.0005
15	**97.92**	97.05	97.47	96.56	97.25
25	98.40	97.34	92.73	**98.78**	98.65
50	96.65	95.50	89.49	97.09	**99.17**
75	94.82	96.07	94.30	**96.75**	95.76
100	96.67	96.18	97.33	96.68	**98.51**
w/o diffusion	98.42	94.36	83.33	**98.75**	97.39

**Table A5:** HTER obtained by evaluating the best models of each alpha (from Table A4) on the test set (the overall lowest HTER is highlighted in bold).

Alpha	15	25	50	75	100	Without Diffusion
HTER (%)	6.25	**1.73**	2.0	6.97	2.97	3.75

**Table A6:** Highest validation accuracy (best model) obtained for different values of learning rate by validating on diffused validation images created with each alpha, during training with Replay-Mobile dataset in CNN-LSTM (the highest validation accuracy for each alpha, and without diffusion, is highlighted in bold).

Alpha	Learning Rate				
	0.001	0.002	0.005	0.0008	0.0005
15	**97.81**	95.89	96.83	96.57	97.73
25	**98.26**	95.91	97.00	97.34	97.40
50	97.40	95.04	93.31	97.27	**98.08**
75	**98.59**	96.39	97.31	98.03	97.34
100	**98.91**	96.71	95.11	97.70	97.01
w/o diffusion	**98.97**	97.08	88.95	97.88	97.42

**Table A7:** Highest validation accuracy (best model) obtained for different values of learning rate by validating on diffused test images created with each alpha, during training, with Replay-Mobile dataset in CNN-LSTM (the highest validation accuracy for each alpha, and without diffusion, is highlighted in bold).

Alpha	Learning Rate				
	0.001	0.002	0.005	0.0008	0.0005
15	93.69	**97.07**	94.40	94.50	95.50
25	93.69	95.28	92.33	**95.33**	94.69
50	93.16	94.67	**95.43**	93.71	92.67
75	95.28	92.67	90.23	**96.01**	94.88
100	93.71	94.95	90.78	94.74	**94.17**
w/o diffusion	93.26	94.04	92.38	92.76	**95.53**

**Table A8:** HTER obtained by evaluating the best models of each alpha (from Table A7) on the test set (the overall lowest HTER is highlighted in bold).

Alpha	15	25	50	75	100	Without Diffusion
HTER (%)	**3.47**	5.83	4.97	4.68	5.77	5.06
